# Use of an innovative cuff pressure control and subglottic secretions drainage system in COVID-19 ARDS patients undergoing pronation

**DOI:** 10.1186/s13054-022-04225-4

**Published:** 2022-11-04

**Authors:** Eloisa Sofia Tanzarella, Gianmarco Lombardi, Silvia Baroni, Francesca Sarlo, Salvatore Lucio Cutuli, Simone Carelli, Melania Cesarano, Veronica Gennenzi, Gabriele Pintaudi, Joel Vargas, Antonio Maria Dell’Anna, Domenico Luca Grieco, Andrea Urbani, Massimo Antonelli, Gennaro De Pascale

**Affiliations:** 1 Dipartimento di scienze dell’emergenza, anestesiologiche e della rianimazione, Fondazione Policlinico Universitario A. Gemelli IRCCS, Rome, Italy; 2 Dipartimento di scienze biotecnologiche di base cliniche intensivologiche e perioperatorie, Fondazione Policlinico Universitario A. Gemelli IRCCS, Rome, Italy; 3 Università Cattolica del Sacro Cuore, Rome, Italy

**Keywords:** Acute respiratory distress syndrome, Ventilator-associated pneumonia, Continuous cuff pressure control, Subglottic secretion drainage, Microaspiration

## Abstract

We conducted a proof of concept study where Anapnoguard endotracheal tubes and its control unit were used in 15 patients with COVID-19 acute respiratory distress syndrome. Anapnoguard system provides suction, venting, rinsing of subglottic space and controls cuff pressure detecting air leakage through the cuff. Alpha-amylase and pepsin levels, as oropharyngeal and gastric microaspiration markers, were assessed from 85 tracheal aspirates in the first 72 h after connection to the system. Oropharyngeal microaspiration occurred in 47 cases (55%). Episodes of gastric microaspiration were not detected. Patient positioning, either prone or supine, did not affect alpha-amylase and pepsin concentration in tracheal secretions. Ventilator-associated pneumonia (VAP) rate was 40%. The use of the AG system provided effective cuff pressure control and subglottic secretions drainage. Despite this, no reduction in the incidence of VAP has been demonstrated, compared to data reported in the current COVID-19 literature. The value of this new technology is worth of being evaluated for the prevention of ventilator-associated respiratory tract infections.

## Introduction

Microaspiration of secretions contaminated with oropharyngeal and gastric pathogens is observed in up to 88% of mechanical ventilated intensive care unit (ICU) patients, representing a key-factor in the pathogenesis of ventilator-associated pneumonia (VAP) [1, 2]. This phenomenon can be identified using pepsin and alpha-amylase dosage in tracheal secretions, with thresholds set at 1685 UI/mL and 200 ng/mL, respectively [3].

Among the strategies developed to prevent microaspiration, many efforts have been focused to optimize subglottic secretion drainage and continuous cuff pressure control.

Subglottic secretions are collected in the space between endotracheal tube cuff and vocal cords. They represent a bacterial reservoir and keeping this space tidy appears to be a useful strategy to reduce the incidence of microaspiration [4].

Likewise, cuff pressure control allows a significant reduction in overinflation and underinflation of the tracheal cuff, recognized risk factors for mucosal damage and microaspiration of contaminated secretions, respectively.

In a previous pilot randomized trial, we showed that a new integrated airway system (AnapoGuard-100 ET and control unit) was safe and effective in terms of cuff pressure control and subglottic secretions evacuation, with a strong trend in reducing microbiologically confirmed VAP [5].

We designed this proof of concept study to assess the rate of microaspiration, defined by the dosage of alpha-amylase and pepsin in tracheal secretions, collected from patients with COVID-19 ARDS undergoing pronation.

## Materials and methods

Fifteen patients with COVID-19 ARDS were intubated with AnapnoGuard (AG) polyvinyl chloride ETs (ID 8.0/7.5 mm), after failing noninvasive ventilatory support. The number of 15 was a convenience sample size, patients were included from 21 October to 14 December 2021. All patients underwent invasive mechanical ventilation with a positive end-expiratory pressure (PEEP) of at least 5 cmH_2_O; oral hygiene with chlorhexidine, daily evaluation for weaning and bed elevation of 30° during supine positioning were observed as a VAP prevention bundle. No selective digestive decontamination policy was applied, whereas all patients had a nasogastric tube and were fed with continuous enteral nutrition.

ETs are equipped with a thin wall polyurethane (50 micron), ellipsoidal shape, cuff; the outer diameter is slightly larger than same size standard tubes. After intubation, patients were connected to the AG-100 control unit (Hospitech Respiration LTD). ETs are designed with three lines: two simultaneous suction lines, and an extra line which provides venting and rinsing of subglottic space. This extra line incorporates a high-sensitivity capnograph, which detects carbon dioxide (CO_2_) levels above the cuff with cycles of a few minutes. The user is asked to set upper and lower pressure limits, then the AG-100 control unit adjusts cuff pressure in two layers. The first control layer consists of keeping the target pressure constant, similarly to electronical manometers. With the second control layer, the device automatically adjusts the target pressure based on the CO_2_ levels that correlate to leaks [6].

Samplings of tracheal aspirates in the first 72 h after connection to the AG-100 system were collected every 8 h, centrifuged at 4000 rpm for 5 min and the supernatants stored at − 80 °C until quantitative measurements of alpha-amylase and pepsin. Salivary alpha-amylase activity was calculated by the difference between total and pancreatic amylase activity, assayed by commercially available kits (Atellica, Siemens Healthcare Diagnostics, USA); pepsin was assayed by kit ELISA (Cusabio, China). All tracheal aspirates were considered as positive if the level of salivary alpha-amylase was > 1685 UI/L and of pepsin > 200 ng/mL.

VAP was defined as the presence of a new and persistent infiltrate on chest X-ray associated with two of the following criteria: purulent tracheal aspirates, hyperthermia (T > 38 °C) or hypothermia (T < 36 °C) and peripheral leucocytosis (WBC > 10,000 μ/L) or leucopenia (WBC < 1500 μ/L). A microbiological confirmation was required using mini-broncho-alveolar lavage (miniBAL) ≥ 10^4^ CFU/mL.

The study was performed in accordance with the Declaration of Helsinki and approved by the Ethics Committee of the Fondazione Policlinico “A. Gemelli IRCCS” (reference number ID3141, “Prot.2019-nCoV_ICU”, date of approval 16/04/2020). A written informed consent or proxy consent was waived, due to the observational nature of the study, according to committee recommendations. All data were anonymous and identified with an admission code number.

Data have been reported as absolute and percentage frequencies as for qualitative variables, whilst quantitative data were expressed either as mean ± standard deviation (SD) or median with interquartile range (IQR). Quantitative data distribution had been previously assessed by the Shapiro–Wilk test. Between-groups differences have been assessed either by the nonparametric Mann–Whitney U test. All statistical analyses were performed using MedCalc Statistical Software version 16.4.3 (MedCalc, Ostend, Belgium).

## Results

Baseline characteristics of patients and results are shown in Table 1.

**Table 1:** Baseline, ventilation and outcome details of 15 enrolled patients

Baseline characteristics	
Age	65 [56–76]
Sex (male)	12 (80)
Cardiovascular Disease	2 (13)
COPD	0 (0)
Diabetes	5 (33)
Liver Disease	2 (13)
CKD	1 (7)
Ventilation and airway management during AG connection	
AG connection, days	7 [5–10]
Mean Pcuff value per patient, cmH_2_O	27.7 [27–29.5]
SS volume per patient, mL	170 [150–275]
Mean daily SS volume per patient, mL	31 [21.5–37]
ETI P/F ratio	84 [79–89]
Pronation cycles per patient	4 [2–5]
IMV, days	9 [6–19]
PEEP, cmH20	9 [8–10]
Shock during AG	7 (47)
CRRT during AG	2 (13)
Patients treated with antibiotics during AG	12 (80)
Antibiotic therapy during AG, days	5 [3–8]
Outcome measures	
Mean Alpha-amylase level per patient, IU/L*	1774 [1651.5–3066]
Mean Pepsin level per patient, ng/mL**	15.1 [7.95–22.35]
Oropharyngeal microaspiration*	47 (55)
Gastric microaspiration	0 (0)
Abundant oropharyngeal microaspiration***	9 (60)
VAP (n)	6 (40)
Late VAP (n)	6 (100)
Time between ETI and VAP (days)	8.5 [7.25–9.75]
Extubation	8 (53)
Tracheostomy	4 (27)
Stridor	2 (13)
ICU LOS (days)	16 [13–24]
28-day mortality (*n*)	4 (27)

Categorical variables are expressed in count and percentage; continuous variables are expressed in median and interquartile range

*TA* tracheal aspirate, *CCI* Charlson Comorbidity Index, *COPD* Chronic Obstructive Pulmonary Disease, *CKD* Chronic Kidney Disease, *ETI* Endotracheal Intubation, *IMV* Invasive Mechanical Ventilation, *CMV* Controlled Mechanical Ventilation, *PEEP* Positive End-Expiratory Pressure, *Pcuff* Cuff Pressure, *SS* subglottic secretions, *AG* AnapnoGuard, *CRRT* Continuous Renal Replacement Therapy, *VAP* Ventilator-Associated Pneumonia, *ETI* EndoTracheal Intubation, *ICU* Intensive Care Unit, *LOS* Length of Stay

^*^85 samples ** 75 samples

^***^Abundant microaspiration of oropharyngeal secretions is defined by the presence of alpha-amylase at a significant concentration (> 1685 IU/mL) in more than 30% of TAs

**Fig. 1:**
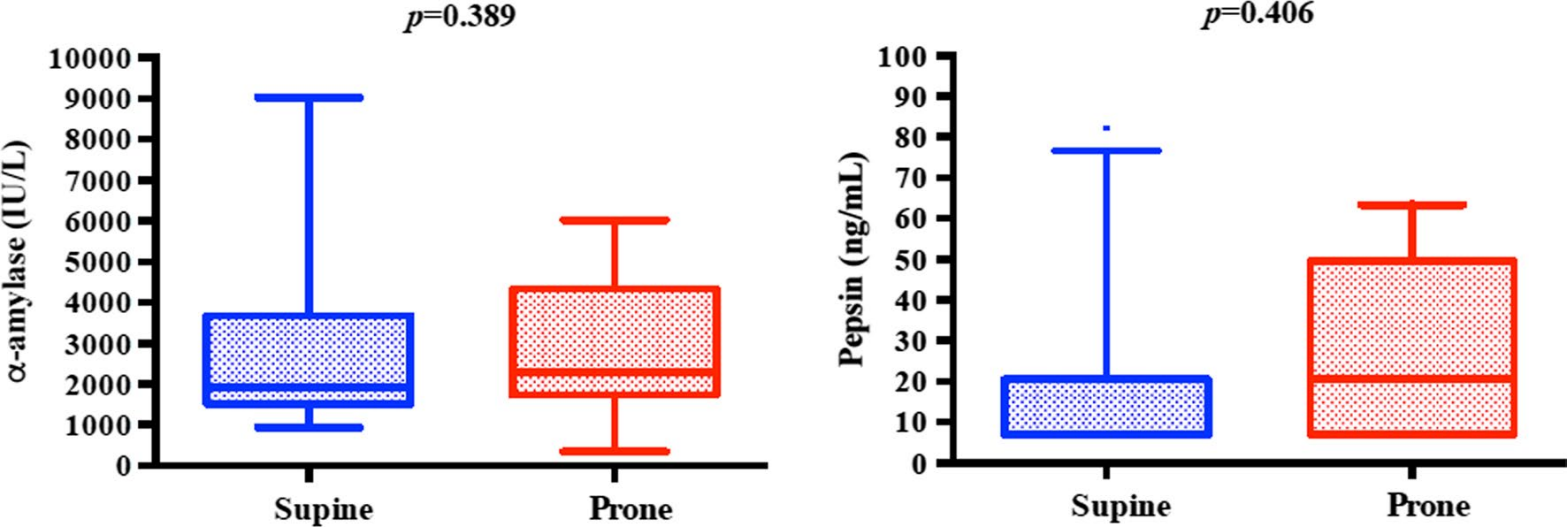
Per patient alpha-amylase and pepsin levels in supine and prone position

Among 15 patients, median age, SAPS-II and SOFA scores were 65 [56–76], 35 [29–44] and 4 [4], respectively. All patients underwent invasive mechanical ventilation, continuous neuromuscular blocking and at least one 18-h pronation cycle. Enteral nutrition was equally delivered during prone and supine positioning (at least 1500 mL per day), without interruption. Alpha-amylase and pepsin were measured in 85 (100%) and 75 (88%) tracheal aspirates, respectively, since a small part of these samples was not quantitatively or qualitatively suitable for detecting pepsin levels. The median number of samplings was 6 [4;7] per patient and 21 (25%) were collected during prone positioning, since most of patients underwent only one 18-h pronation cycle in the predefined sampling time frame. Cuff pressure values were stable between 25 and 30 cmH_2_0 with a median daily subglottic secretions volume of 31 mL. Oropharyngeal microaspiration (alpha-amylase > 1685 IU/L) was diagnosed in 47 TA (55%). Conversely, no tracheal secretions samplings showed evidence of gastric microaspiration (pepsin > 200 ng/mL). There was no correlation regarding alpha-amylase and pepsin levels between prone and supine positioning (2305.6 [1730.9–4331.3] IU/mL versus 1932.5 [1515.5–3673.2] IU/mL; 7.8 [7.8–23.55] ng/mL versus 26.6 [7.8–52.25] ng/mL; *p* = 0.389 and *p* = 0.406), Fig. 1. The incidence of VAP, diagnosed with radiological and clinical criteria and confirmed by a microbiologically documentation, was 40% (27.65 episodes/1000 intubation days), with a median mechanical ventilation time to infection of 8.5 [7.25–9.75] days. Stridor after extubation was observed in two patients. No ET misplacements occurred.

## Discussion

We studied the occurrence of microaspiration in 15 COVID-19 ARDS patients intubated with AG ET and connected to AG-100 control unit.

Previous randomized trials showed the efficacy of automatic cuff pressure control to minimize ET cuff underinflation and microaspiration [5, 7]. However, available electronic or pneumatic devices are not able to identify the optimal value for the individual patient. Conversely, the AG-100 system, detecting CO_2_ leaks above the ET cuff, provides an accurate control of ET cuff pressure, regardless of prone positioning and variations in PEEP level.

Patients’ subglottic secretions intermittent drainage, either manual or automatic, are now considered an effective intervention to reduce VAP [4]. However, standard systems may harbour a suboptimal evacuation capability due to line obstructions or ‘vacuum’ effect occurrence. Due to the presence of a double suction lumen and an extra line for venting and rinsing the subglottic space, in our patients no obstructions were detected, allowing the drainage of relevant amounts of SS (about 30 mL per day).

We observed that this new technology can provide cuff pressure control and subglottic secretions drainage, with good potential to reduce the incidence of oropharyngeal and gastric microaspirations, both in supine and prone position. Current literature shows microaspiration rates ranging from 0 to 50% for gastric content and from 50 to 80% for oropharyngeal content, according to the study population and the adopted preventing measure [3]. Our results showed that neither prone positioning nor deep sedation and neuromuscular blocking agent use was associated with higher alpha-amylase or pepsin levels in TAs, obtaining an overall incidence of oropharyngeal microaspiration of 55%, which was among the lowest data reported in current literature [8]. Additionally, no relevant microaspiration of gastric content was observed, even when patients were kept prone for many hours. The absence of pepsin in TAs may be explained by the enteral nutrition effect on overall dilution of gastric content and on pH-driven pepsin secretion, whilst fluid oropharyngeal secretions are more likely to leak around, thus being detected in tracheal secretions. This issue had already been pointed out by Nseir et al. [9].

Conversely, the incidence of VAP (40%) has not been proven to be inferior to data reported in the current literature.

In fact, as far as is known that patients with SARS-COV-2 infection are more likely to develop ventilator-associated lower respiratory tract infections, the incidence of VAP in a cohort of patients recently investigated by Rouzé et al. was 36.1% [10]. It cannot be excluded that one of the reasons of this poor result, besides the small number of patients, could be the shape and the thinness of the polyurethane cuff (50 micron), that does not completely prevent the migration of subglottic secretions.

Our study has some limitations. Mainly, the small sample size and the observational nature of the design, without a control group, do not allow to generalize our results and to perform comparisons inside the cohort; in addition, no microbiological samples to detect bacterial tracheobronchial colonization were collected. Finally, we cannot exclude that the polyurethan cuff ‘per se’ may have consistently contributed to the observed results.

However, this is the first study where oropharyngeal and gastric microaspiration was investigated in COVID-19 ARDS patients using a new automatic ET cuff pressure control and subglottic secretions drainage, during supine and prone position.

## Conclusions

In COVID-19 ARDS patients undergoing pronation, the use of the AG system, although limiting the amount of oropharyngeal and gastric microaspiration, did not reduce the incidence of VAP compared to current COVID-19 literature. These preliminary results represent a proof of concept to better investigate the efficacy of this new technology for VAP prevention.

## Data Availability

The datasets used and/or analysed during the current study are available from the corresponding author on reasonable request.
